# Supporting the Implementation of Connected Care Technologies in the Veterans Health Administration: Cross-Sectional Survey Findings from the Veterans Engagement with Technology Collaborative (VET-C) Cohort

**DOI:** 10.2196/21214

**Published:** 2020-09-30

**Authors:** Bella Etingen, Daniel J Amante, Rachael N Martinez, Bridget M Smith, Stephanie L Shimada, Lorilei Richardson, Angela Patterson, Thomas K Houston, Kathleen L Frisbee, Timothy P Hogan

**Affiliations:** 1 eHealth Partnered Evaluation Initiative Edith Nourse Rogers Memorial Veterans Hospital Bedford, MA United States; 2 Center of Innovation for Complex Chronic Healthcare Hines Veterans Affairs Hospital Hines, IL United States; 3 Division of Health Informatics and Implementation Science Department of Population and Quantitative Health Sciences University of Massachusetts Medical School Worcester, MA United States; 4 Northwestern University Feinberg School of Medicine Chicago, IL United States; 5 Center for Healthcare Organization and Implementation Research Edith Nourse Rogers Memorial Veterans Hospital Bedford, MA United States; 6 Department of Health Law, Policy, and Management Boston University School of Public Health Boston, MA United States; 7 Office of Connected Care Veterans Health Administration US Department of Veterans Affairs Washington, DC United States; 8 Department of Population and Data Sciences University of Texas Southwestern Medical Center Dallas, TX United States

**Keywords:** eHealth, mobile health, patient engagement, telehealth, veterans

## Abstract

**Background:**

Widespread adoption, use, and integration of patient-facing technologies into the workflow of health care systems has been slow, thus limiting the realization of their potential. A growing body of work has focused on how best to promote adoption and use of these technologies and measure their impacts on processes of care and outcomes. This body of work currently suffers from limitations (eg, cross-sectional analyses, limited patient-generated data linked with clinical records) and would benefit from institutional infrastructure to enhance available data and integrate the voice of the patient into implementation and evaluation efforts.

**Objective:**

The Veterans Health Administration (VHA) has launched an initiative called the Veterans Engagement with Technology Collaborative cohort to directly address these challenges. This paper reports the process by which the cohort was developed and describes the baseline data being collected from cohort members. The overarching goal of the Veterans Engagement with Technology Collaborative cohort is to directly engage veterans in the evaluation of new VHA patient-facing technologies and in so doing, to create new infrastructure to support related quality improvement and evaluation activities.

**Methods:**

Inclusion criteria for veterans to be eligible for membership in the cohort included being an active user of VHA health care services, having a mobile phone, and being an established user of existing VHA patient-facing technologies as represented by use of the secure messaging feature of VHA’s patient portal. Between 2017 and 2018, we recruited veterans who met these criteria and administered a survey to them over the telephone.

**Results:**

The majority of participants (N=2727) were male (2268/2727, 83.2%), White (2226/2727, 81.6%), living in their own apartment or house (2519/2696, 93.4%), and had completed some college (1176/2701, 43.5%) or an advanced degree (1178/2701, 43.6%). Cohort members were 59.9 years old, on average. The majority self-reported their health status as being good (1055/2725, 38.7%) or very good (524/2725, 19.2%). Most cohort members owned a personal computer (2609/2725, 95.7%), tablet computer (1616/2716, 59.5%), and/or smartphone (2438/2722, 89.6%).

**Conclusions:**

The Veterans Engagement with Technology Collaborative cohort is an example of a VHA learning health care system initiative designed to support the data-driven implementation of patient-facing technologies into practice and measurement of their impacts. With this initiative, VHA is building capacity for future, rapid, rigorous evaluation and quality improvement efforts to enhance understanding of the adoption, use, and impact of patient-facing technologies.

## Introduction

### Background

Health care systems are facing an era of unprecedented growth in the number of patient-facing eHealth technologies available. Personal health record portals, mobile health (mHealth) apps, clinical videoconferencing platforms, automated texting systems, and other such tools have great potential to reach, engage, and empower patients, to support access to and delivery of care, and to improve outcomes; however, this potential has not yet been widely realized [1-4]. The experiences of many health care organizations underscore that promoting patient adoption and use of these technologies is difficult, and their effective integration into routine care can be elusive. Different patients may have different levels of interest in using eHealth technologies and may choose to abandon use if their expectations are not sufficiently met [5]. Because these technologies have, in many cases, not yet attained widespread recognition or endorsement among health care providers, patient awareness of their availability may be limited [6]. Similarly, the extent to which patient-facing eHealth technologies fit into a patient’s daily life and their larger technological milieu can also directly affect their perceived usefulness and benefits.

Recognizing the importance of these issues, a growing body of work has focused on how best to bolster patient adoption and use of eHealth technologies, monitor their use, and measure their impacts on processes of care and outcomes [2,7,8]. This work, however, has also faced challenges. Particularly, studies that have relied on secondary data (eg, hospital administrative data or technology activity log data) to measure exposure to and use of patient-facing technologies and their effect on health care utilization and outcomes have encountered other issues, the most problematic perhaps being missing data. Many covariates related to technology use (eg, health literacy, education level, income level) are simply not available through such data sources. However, these covariates are essential to understanding technology adoption and adjusting for confounding factors when modeling associations with outcomes. A similar challenge exists for outcomes of interest to health care organizations, including patient-reported outcomes and perceptions of care. These challenges speak to how important it is for health care organizations to consider developing infrastructure capable of offering more complete data and placing the voice of the patient in the foreground as the key stakeholder in efforts to implement and evaluate patient-facing eHealth technologies [2].

### Transforming Care Through eHealth Technologies in the Veterans Health Administration

The Veterans Health Administration (VHA) has been a pioneering, national leader in developing patient-facing eHealth technologies and integrating their use into clinical practice. Similar to other health care organizations, the VHA has developed a range of such technologies intended for use by the patient population that they serve—veterans of the US military. Table 1 presents key categories of these technologies and a description of each.

**Table 1:** Select VHA patient-facing eHealth technologies [9].

Technologies		Description
**Key telehealth services**		
	Remote patient monitoring	Health data (eg, blood pressure, weight, glucose level) is gathered by a device issued to the patient and that data is, in turn, sent to the patient’s care team.
	Video appointments	Camera and audio on smartphones, computers, tablets, and other devices are used to support a video appointment between a patient and their care team from the comfort of the patient’s home.
Personal health record portal		VHA’s^a^ tethered patient portal offers health education resources and features that support transactions with the health care system (eg, medication refilling), communication between a patient and their VHA clinical team members (eg, asynchronous secure messaging), self-management support (eg, tracking behaviors and symptoms), and access to the content of the patient’s medical record (eg, Blue Button).
Automated text-messaging		VHA’s protocol-driven automated text-messaging system provides tailored support for condition-specific self-management and other health behaviors through one-way and two-way messaging.
Mobile apps		VHA has developed a suite of mobile apps (which are designed to address the unique needs of the Veteran population) intended to promote wellness and healthy behaviors, provide condition-specific self-management support, support other transactions with the health care system, and enhance clinical management.

^a^VHA: Veterans Health Administration.

In addition to those presented in Table 1, new VHA patient-facing technologies are in continual development. As these technologies have been developed and rolled out at different times and are intended to meet different needs, the extent of their adoption within the veteran population varies considerably [10-12]. The work of developing and implementing these technologies is the responsibility of VHA’s Office of Connected Care, which oversees VHA’s digital health strategy and is focused on improving VHA care through technology that engages veterans beyond traditional health care visits.

Recognizing the need for further insights to advance the implementation and evaluation of their portfolio of technologies, in 2016, the VHA Office of Connected Care, in conjunction with the VHA Quality Enhancement Research Initiative Program, funded an effort to recruit a group of veterans willing to make a long-term commitment to providing feedback on the latest VHA patient-facing technologies and helping the VHA understand their potential benefits. Branded the Veterans Engagement with Technology Collaborative, the overarching goal of this cohort is to directly engage veterans in the evaluation of new VHA patient-facing technologies intended to improve access to care, enhance care coordination, and support self-management, and in so doing, to create new infrastructure to support related research and evaluation activities. Soliciting the expertise of patients and integrating their perspectives into VHA’s technology evaluation efforts demonstrates the values of participatory medicine, a patient-centered philosophy, in action. It also aligns with broader initiatives by VHA and its leadership to enhance veteran engagement in an effort to realize health care system improvements that resonate with the veteran population.

Importantly, the development and use of the Veterans Engagement with Technology Collaborative cohort was designed to meet the criteria for quality improvement and was subsequently reviewed by the Institutional Review Board at the Edith Nourse Rogers Memorial Veterans Hospital in Bedford, Massachusetts, and determined to be such [13]. This designation is an important step toward realizing the National Academy of Medicine’s vision for learning health systems, where “science, informatics, incentives, and culture are aligned for continuous improvement and innovation, with best practices seamlessly embedded in the delivery process [14].”

### Objectives

The specific objectives of the Veterans Engagement with Technology Collaborative cohort were to (1) identify the extent of exposure and use, as well as patient perceptions, of select VHA patient-facing eHealth technologies, (2) understand characteristics and determinants associated with adoption and use of specific VHA patient-facing eHealth technologies, and (3) examine the impact of patient-facing eHealth technology use on select patient-reported outcomes, experience with and perceptions of VHA care.

This paper reports the process by which the Veterans Engagement with Technology Collaborative cohort was developed, describes the baseline cross-sectional survey data collected from cohort members, and details future plans for longitudinal follow-up and cohort maintenance.

## Methods

### Design

The Veterans Engagement with Technology Collaborative cohort is a longitudinal cohort comprised of survey data collection across multiple time-points.

### Participants

Individuals were eligible for inclusion in the cohort if they were a veteran of the US military and an active user of VHA health care services. In addition, because we aimed to enroll veterans who were users of patient-facing eHealth technology, at the time of screening, all veterans invited to be in the cohort were required to have a mobile phone and have sent at least 5 (but less than 30, which comprised the cut-off for the 95th percentile of secure message volume) secure messages using the patient-to–clinical team secure messaging feature of the VHA personal health record portal in the year prior to recruitment.

### Setting

The VHA health care system provides health care and other benefits (such as compensation or pension, life insurance, and vocational rehabilitation) to approximately 9.7 million veterans of the US military, which is nearly half of the entire veteran population [15]. On average, compared to civilians, veterans are older, predominantly male, and experience more health concerns, and compared to veterans who do not receive VHA care, veterans who use VHA services tend to be sicker and have less income [16]. The veterans who currently comprise the Veterans Engagement with Technology Collaborative cohort received care from at least 1 of 14 purposefully sampled VHA medical centers across the United States and include residents of most states across both urban and rural settings. In addition to securing a diverse geographic representation of veterans, as well as adequate representation of women and racial or ethnic minority veterans, we selected these 14 facilities based on several criteria deemed important to the overall goals of the Veterans Engagement with Technology Collaborative cohort. Criteria included engaging veterans seen for care at VHA facilities representing different geographic regions of the country that had high rates of adoption of the VHA’s personal health record portal secure messaging feature, a track record of being a site of early adoption for other VHA patient-facing eHealth technologies (eg, VHA’s automated text messaging system, video-to-home telehealth, online scheduling), and plans for implementing other VHA eHealth technologies in the future. Veterans who met these criteria were included on recruitment lists, which detailed their name and contact information (eg, telephone number) as listed in VHA administrative records housed in the VHA Corporate Data Warehouse.

### Recruitment

In order to recruit veterans into the cohort, we provided evaluation team members with these recruitment lists, and team members reached out to each veteran by phone to invite them to participate. We called all eligible patients one time unless they requested a call back, in which case, we made one additional follow-up call. During the call, team members read a script to the veterans explaining the purpose of the Veterans Engagement with Technology Collaborative cohort and their eligibility to participate. Evaluation team members also explained the long-term commitment requested of each participating veteran. That is, veterans who consented to participate in the cohort during this recruitment call would be engaged in ongoing survey efforts and periodic evaluation activities over time. If the veteran agreed to participate, the evaluation team member then collected the baseline survey data. Responses were entered in real time, into a secure electronic database system (REDCap, Research Electronic Data Capture; Vanderbilt University). Survey administration took approximately 20 minutes per patient. All recruitment calls and survey data collection efforts occurred in the 2017 calendar year. No incentives were provided at baseline data collection.

### Baseline Data Collection

#### Survey Measures

The Veterans Engagement with Technology Collaborative baseline survey was used to gather patient-level information that is not readily available in clinical or administrative databases, such as patient perceptions of access to and current use of health care and patient-facing technologies, perceptions of health care team member support of these technologies, patient-provider communication, and sociodemographic information such as health literacy and financial status.

We asked participants to report on factors associated with their health and health care use, including whether they usually receive health care from the VHA, from outside of the VHA, or both, and how long it takes to travel from their home to their VHA primary care doctor’s office. We also asked them whether anyone helps them manage their health or health care, how they perceive their overall health status [17], and the extent to which they adhere to taking their prescribed medications [18].

In addition, several questions addressed health-related goal setting behaviors; specifically, participants were asked, “In the last 6 months, did anyone in your VHA provider’s office talk with you about specific goals for your health?” and “In the past 6 months, have you set any goals related to your health?” Those who set a goal were asked, “What health-related goal or goals have you made in the past 6 months?”; “Have you been able to achieve this health-related goal?”; and “Have you used an app on a smartphone or tablet to help you achieve/work on this health-related goal?”

#### Technology Ownership and Use

Technology ownership and use questions assessed personal computer ownership, tablet computer ownership, mobile phone ownership, whether participants ever borrow any of these devices from others, and whether participants ever use devices to measure and send health information (eg, blood pressure, blood glucose level, weight) to their care providers. We also asked participants whether they like to be among the first to get a new device, tech gadget, or app when it comes out (ie, do they consider themselves early adopters), whether they use social media (ie, Twitter, Facebook, Instagram, Pinterest) [19], and how comfortable or confident they feel using computers on a scale of 0 (not at all) to 5 (very) [20].

We used adapted items from the VHA Survey of Healthcare Experience of Patients to assess participant perceptions of their health and health care–related communication, including how easy it is for them to communicate with their care providers when needed on a scale of 1 (very easy) to 5 (very difficult), how often (in the prior 6 months) they received a response within 1 day when they needed to communicate with their care provider’s office on a scale of 1 (never) to 4 (always), and how often (in the prior 6 months) they received a response from their care provider’s office as soon as they needed it when they contacted the office after hours on a scale of 1 (never) to 4 (always).

We also asked participants how big of a problem on a scale of 1 (very big problem) to 5 (not a problem) each of the following are for them: poor communication between different doctors or clinics, disagreements between their doctors about their diagnosis or the best treatment for them, and having their concerns ignored or overlooked by their health care providers. In addition, we asked participants to report how confident they feel filling out medical forms by themselves on a scale of 1 (not at all) to 5 (extremely) [21], and how easy or hard they find it to understand medical statistics on a scale of 1 (very easy) to 4 (very hard) [22].

Additionally, we asked participants to report their agreement on a scale of 1 (strongly disagree) to 7 (strongly agree) with a number of statements about secure messaging: their health care team encourages them to ask questions using secure messaging; in secure messaging, their health care team answers their questions related to their health fully and carefully; and the secure messages they receive make them feel that their health care team cares about them as a person.

We collected demographic information including age, gender, race, ethnicity, relationship status, highest level of education achieved, living arrangement, and financial difficulty.

#### Data Linked From VHA Records

We obtained data on chronic health conditions and information used to calculate a Hierarchical Condition Community [23,24] score for the veterans in our cohort from the VHA Corporate Data Warehouse. Hierarchical Condition Community scores represent a comorbidity index that takes into account an individual’s age, gender, medical diagnoses, and eligibility for Medicare and Medicaid services [23,24]. Typically, the range of Hierarchical Condition Community scores is between 0.9 and 1.7; scores less than 1 are often interpreted as healthy [25].

### Statistical Analyses

We will use the data provided by the cohort to examine novel and important issues related to technology use among veterans, including perceptions of newly developed patient-facing technologies, impacts of use on perceptions of and satisfaction with care delivery, and associations with important health and utilization outcomes (eg, health-related goal setting and attainment, medication adherence, communication with care team members). In this manuscript, we examine frequencies of responses to key survey items. Statistical analyses were conducted using STATA (version 14.2; StataCorp LLC).

## Results

Responses to survey items intended to characterize the sample and gather information on covariates are presented below in the narrative and accompanying tables.

### Response Rate and Cohort Derivation

We identified and attempted to contact 20,091 veterans who met inclusion criteria for the cohort. Of these veterans, 5877 were reached by phone, 2735 agreed to participate, and 2727 completed the survey (46.4% participation rate).

### Demographics

The veterans who comprised the cohort were 59.9 years old, on average, at the time that the first survey was administered in 2017. Participants were predominantly male (2268/2727, 83.2%), White (2226/2727, 81.6%), and living in their own apartment or house (2519/2696, 93.4%). Most had completed some college (1176/2701, 43.5%) or an advanced degree (1178/2701, 43.6%) and were married or in a civil union (1734/2687, 64.5%). Most (1813/2637, 68.8%) reported that it was not very difficult for them to pay for basics like food and heating or cooling (Table 2).

**Table 2:** Demographics.

Variable		Value
**Age (years) (N=2727)**		
	mean (SD)	59.9 (13.1)
	range	24.5-95.8
**Gender (N=2727), n (%)**		
	Male	2268 (83.2)
	Female	459 (16.8)
**Race (N=2727), n (%)**		
	White	2226 (81.6)
	Black or African American	317 (11.6)
	Asian	13 (0.5)
	Native Hawaiian or other Pacific Islander	6 (0.2)
	American Indian or Alaskan Native	63 (2.3)
	Other	86 (3.2)
	Declined to answer	56 (2.1)
**Ethnicity (N=2727), n (%)**		
	Yes, Hispanic or Latino	135 (5.0)
	No, not Hispanic or Latino	2592 (95.1)
**Relationship status (n=2687), n (%)**		
	Married or in a civil union	1734 (64.5)
	Neither married, nor in a civil union^a^	953 (35.5)
**Education status (n=2701), n (%)**		
	High school graduate or less	347 (12.9)
	At least some college or vocational school (1-4 years)	1176 (43.5)
	Master’s, professional, or doctoral degree	1178 (43.6)
**Living arrangement (n=2696), n (%)**		
	Own apartment or house	2519 (93.4)
	Friend or relative’s apartment or house	120 (4.5)
	Other^b^	57 (2.1)
**Financial difficulty^c^ (n=2637), n (%)**		
	Not very hard	1813 (68.8)
	Somewhat hard, hard, or very hard	824 (31.3)

^a^Defined as engaged or in a relationship, single, separated, divorced, or widowed.

^b^Defined as school or dormitory, hospital or detox center, nursing home or assisted living, car or street, or jail or prison.

^c^Based on response to the question “How hard is it for you (and your family) to pay for the very basics like food and heating/cooling?”

### Health and Health Care Use

The most prevalent chronic conditions among this sample were hypertension (1699/2727, 62.3%), osteoarthritis (1444/2727, 53.0%), and depression (1109/2727, 40.7%). Most participants reported that they receive their health care mostly at the VHA (2142/2718, 78.8%), and nearly half (1326/2720, 48.8%) reported living less than 30 minutes away from the VHA at which they received primary care. The majority of the cohort reported being in good (1055/2725, 38.7%) or very good (524/2725, 19.2%) health and that they always take their medications as recommended by their care providers (2273/2698, 84.3%) (Table 3).

**Table 3:** Health and health care use.

Variable		Value
**Chronic conditions^a^ (N=2727), n (%)**		
	Acute myocardial infarction	297 (10.9)
	Atrial fibrillation	227 (8.3)
	Heart failure	206 (7.6)
	Ischemic heart disease	646 (23.7)
	Peripheral vascular disease	352 (12.9)
	Hypertension	1699 (62.3)
	Asthma	575 (21.1)
	Breast cancer	23 (0.8)
	Colorectal cancer	35 (1.3)
	Prostate cancer	105 (3.9)
	Lung cancer	32 (1.2)
	Endometrial cancer	2 (0.1)
	Chronic kidney disease	646 (23.7)
	Chronic obstructive pulmonary disease	105 (3.9)
	Depression	1109 (40.7)
	Diabetes	1024 (37.6)
	Osteoarthritis	1444 (53.0)
	Stroke	160 (5.9)
	Posttraumatic stress disorder	773 (28.4)
	Anxiety	705 (25.9)
	Traumatic brain injury	205 (7.5)
**Health status (general) (n=2725), n (%)**		
	Excellent	144 (5.3)
	Very good	524 (19.2)
	Good	1055 (38.7)
	Fair	797 (29.3)
	Poor	205 (7.5)
**Hierarchical Condition Community score^a^ (N=2727), n (%)**		
	Mean (SD)	0.3 (0.03)
	Range	0.3-0.4
**Health care receipt (n=2718), n (%)**		
	Mostly at the VHA^b^	2142 (78.8)
	Mostly outside VHA	159 (5.9)
	About half in VHA, half outside VHA	417 (15.3)
**Travel time (to VHA primary care doctor’s office) (n=2720), n (%)**		
	<30 minutes	1326 (48.8)
	31 to 60 minutes	970 (35.7)
	>60 minutes	424 (15.6)
**Assistance in managing health or health care (N=2727), n (%)**		
	Paid caregiver	92 (3.4)
	Spouse/partner	694 (25.5)
	Children	157 (5.8)
	Family or extended family member	176 (6.5)
	Friend	100 (3.7)
	Other	29 (1.1)
	N/A^c^	1717 (63.0)
**Medication adherence^d^ (n=2698), n (%)**		
	All (100%) of the time	2273 (84.3)
	Not all of the time	425 (15.8)

^a^In the prior five years.

^b^VHA: Veterans Health Administration.

^c^N/A: not applicable.

^d^Based on response to the question “In the past month, how often did you take your medications as the doctor prescribed?”

### Technology Ownership and Use

The majority of participants reported owning a personal computer (2609/2725, 95.7%), tablet computer (1616/2716, 59.5%), or smartphone (2438/2722, 89.6%). Most (2412/2727, 88.5%) reported that they do not borrow technological devices from others. Most reported that they agree (669/2715, 24.6%) or strongly agree (813/2715, 29.9%) that they are an early adopter of new technology and that they are very comfortable or confident using computers (1878/2705, 69.4%) (Table 4).

**Table 4:** Technology ownership and use.

Variable		Value
**Owns a desktop or laptop computer (n=2725), n (%)**		
	Yes	2609 (95.7)
	No	116 (4.3)
**Owns a tablet computer (iPad, Kindle Fire, etc) (n=2716), n (%)**		
	Yes	1616 (59.5)
	No	1100 (40.5)
**Mobile phone ownership^a^ (n=2722), n (%)**		
	Smartphone^b^	2438 (89.6)
	Nonsmartphone mobile phone	261 (9.6)
	No mobile phone	23 (0.8)
**Borrow devices from others (N=2727), n (%)**		
	Sometimes use friend’s device	48 (1.8)
	Sometimes use family member’s device	167 (6.1)
	Use device at work	77 (2.8)
	Sometimes use library/senior center/hospital/other location’s device	42 (1.5)
	No	2412 (88.5)
**Use of devices to measure and send health measurements to health care team (n=2717), n (%)**		
	Yes	679 (25.0)
	No	2038 (75.0)
**Early tech adopter (n=2715), n (%)**		
	Strongly agree	813 (29.9)
	Agree	669 (24.6)
	Neutral	692 (25.5)
	Disagree	323 (11.9)
	Strongly disagree	218 (8.0)
**Social media use (N=2727), n (%)**		
	Twitter	552 (20.2)
	Facebook	1955 (71.7)
	Instagram	466 (17.1)
	Pinterest	479 (17.6)
**Comfort or confidence using computers (n=2705), n (%)**		
	Very comfortable or confident	1878 (69.4)
	Less than very comfortable or confident	827 (30.6)

^a^Based on response to the statement “If you have multiple cell phones, select the one you use most often.”

^b^iPhone, Android, Blackberry, Windows phone, Symbian, or some other type of smartphone.

## Discussion

### Veterans Engagement With Technology Collaborative Cohort and the Learning Health Care System

Evidence regarding the use and effectiveness of patient-facing technologies is accumulating [26-31], but considerable gaps remain. Given the abundance of new patient-facing technologies that are being (and will continue to be) developed, health care systems will face an ongoing challenge to determine if and how best these technologies can be used to support patients and improve health care quality. The concept of the learning health care system holds that “learning while doing” should be the penultimate goal of health care organizations and emphasizes the importance of appropriate infrastructure, data resources, and partnerships between stakeholders [32,33]. The learning health care system is predicated on the active collaboration among all participants in a system, underscoring how critical it is to engage stakeholders—including patients—in evaluation and implementation efforts [33]. What we have described in this paper is one initiative that is helping to accentuate the voice of the veteran in ongoing efforts to realize the vision of the learning health care system within VHA.

The Veterans Engagement with Technology Collaborative cohort directly engages veterans to understand the potential benefits and possible unintended consequences related to the patient-facing technologies that the VHA is developing and implementing. This new initiative provides a means for veterans and program evaluators to test these technologies on a timeline that more closely reflects their rapid development and evolution. It also supports the rapid evaluation of unexpected but significant changes in the health care system that may influence the role of technology in care delivery. The coronavirus disease 2019 (COVID-19) pandemic and the surge in patient-facing technology use that has accompanied it, is a case in point. The cohort reflects a strong partnership between operational entities within a large health care system and established members of its research and evaluation community to develop new infrastructure to support the broad goal of implementing and measuring the impacts of patient-facing eHealth technologies in practice.

The application and advancement of health- and health care–related technology has the potential to help revolutionize care, improve patient outcomes and satisfaction, and reduce health care costs [34]. Notably, the veterans described in this manuscript have made a commitment to participate in longitudinal follow-up, which will consist of follow-up survey efforts by our team over several years, the content and timing of which will be driven by the VHA’s evaluation needs. Some question items and scales included on the baseline survey will be repeated, thus providing longitudinal data. We expect that other question items and scales will be added based on emergent priorities. Through our follow-up data collection efforts, this cohort of veterans will also serve as a resource to evaluate future technologies, such as new mobile health apps developed for use within the VHA to optimize important outcomes (eg, access to and coordination of services, patient activation and self-management, goal setting and attainment). Cohort member involvement in evaluations is likely to include providing feedback via multiple approaches including targeted surveys and interviews focused on user experiences. Evaluation activities that incorporate the voice of the veteran are increasingly recognized by the VHA health care system and its leadership as powerful approaches to improving health care delivery in ways that reflect the needs and preferences of the veteran population. The Veterans Engagement with Technology Collaborative cohort aligns with other veteran engagement initiatives currently being implemented to improve the policy and patient relevance of VHA research and evaluation activities.

### Limitations

Survey responses are subject to a number of biases (eg, recall bias, response bias), and the cross-sectional design does not allow us to determine causal relationships. Furthermore, the veterans in our sample represent a subset of the veteran population and are known technology users, and in comparison to the general population of veterans who use VHA health care, are approximately one year younger, on average [35], and include a greater proportion of women [36], individuals who are White and of non-Hispanic ethnicity, individuals who report being in fair or poor health [35], and individuals who have health conditions such as depression [37,38], diabetes [39], posttraumatic stress disorder [38,40], hypertension [41], and anxiety [38]. These differences may impact patient-facing eHealth technology preferences or use and limit the generalizability of the data collected thus far, as well as findings from future evaluations conducted with this cohort.

We also acknowledge that the proxy indicators used for technology adoption (ie, veteran use of secure messaging in the prior year, early implementation of eHealth technologies at VHA facilities where veterans receive health care) do not comprehensively reflect all factors that may impact use of patient-facing eHealth technologies. Future work may consider additional factors, for example, aspects of the technology’s design, usability, and utility. In addition, while we recognize the importance of health care providers and their perspectives in the development and evaluation of patient-facing technologies, we have not yet incorporated their perspectives into the initiative. Because all patient-facing technologies have reciprocal repercussions for the health care team members of patients who use them, in the future, we also plan to assess the perspectives of VHA health care team members.

### Conclusions

Through the development of the Veterans Engagement with Technology Collaborative cohort, the VHA is laying the foundation for future, rapid, rigorous evaluation and quality improvement efforts that can advance our understanding of the adoption, use, and impact of patient-facing technologies and inform related policy decisions and funding priorities. The development and maintenance of the Veterans Engagement with Technology Collaborative cohort over time establishes a diverse group of veterans who can test emerging VHA patient-facing technologies and technology-based interventions. This infrastructure will help us obtain early feedback on these technologies, as well as advance our understanding of whether certain groups of veterans require extra support to adopt these technologies and use them over time.

## Abbreviations

mHealth: mobile health
VHA: Veterans Health Administration
